# Effect of preoperative keratometry on visual outcomes after small-incision lenticule extraction for myopia

**DOI:** 10.1007/s10792-021-02167-4

**Published:** 2022-01-08

**Authors:** Seongjun Lee, Sinwoo Bae, Moonsun Jung

**Affiliations:** 1 Nuri Eye Hospital, 61 Dunsan-ro, Daejeon, Seo-gu 35233 South Korea; 2 Chungbuk National University Hospital, 776, 1 Sunhwan-ro, Seowon-gu, Cheongju, Chungcheongbuk-do 28644 South Korea

**Keywords:** Small incision lenticule extraction (SMILE), Preoperative keratometry, Postoperative refraction

## Abstract

**Purpose:**

To investigate the relationship between preoperative keratometry (K) and postoperative refraction and compare the visual outcomes after small-incision lenticule extraction (SMILE) between preoperative flat and steep corneas.

**Methods:**

This study involved 814 consecutive eyes of 409 patients who underwent SMILE. A month later, a linear regression analysis of the relationship between preoperative K and the residual spherical equivalent (SE) along with eyes divided by a single standard deviation between flat and steep corneas (< 41.85 *D*, > 44.57 *D*, respectively) was conducted. Eyes were distinguished based on the degree of myopia.

**Results:**

One month after surgery, no significant correlation existed between mean preoperative K and residual SE (*P* = 0.459). Linear regression analysis showed a weak negative correlation between flat corneas (*r*^2^ = 0.042, *P* = 0.025) rather than steep corneas (*P* = 0.908). Eyes with preoperative low myopia (< 3.00 D) (*r*^2^ = 0.233, *P* = 0.001) had a weak correlation compared with moderate and high myopia (*P* = 0.272, *P* = 0.257, respectively). Twelve months later, the predictability, safety, and efficacy did not vary between preoperative flat and steep corneas (*P* > 0.05).

**Conclusions:**

One month after SMILE for myopia, the corneas were flatter in the preoperative flat corneas or all the low myopic corneas, and they were more overcorrected. However, preoperative corneal curvature does not influence visual outcomes at 1 year after SMILE.

## Introduction

Even though small-incision lenticule extraction (SMILE) is a safe, efficient, and predictable surgical method for correction of myopia, the predictability of the technique as well as laser in situ keratomileusis (LASIK) and surface ablation is disputed, especially in eyes with high degrees of myopia or hyperopia [1–9]. To date, a few studies have evaluated the effect of preoperative keratometry on visual outcomes after LASIK or surface ablation and predictors of SMILE outcomes [10–13].

Previous studies showed no relationship between preoperative corneal curvature and postoperative predictability after myopic laser-assisted subepithelial keratectomy (LASEK) [14]; however, when treating preoperative higher myopia in steeper corneas, a weak tendency existed toward overcorrection [12]. In previous studies evaluating the effect of preoperative keratometry (K) on visual outcomes after LASIK for myopia, eyes with flatter corneas tended to have greater undercorrection than eyes with similar myopia and steeper corneas, and undercorrection and loss of best spectacle-corrected visual acuity following hyperopic LASIK in eyes with steep corneas [10, 11]. Besides, moderately myopic eyes with flatter corneas preoperatively show better visual prognosis following LASIK compared with steeper corneas [15]. Because the clinical outcome of refractive surgery depends, at least in part, on the type of corneal repair response induced by the treatment, it is of considerable interest to understand the molecular and cellular events leading to the formation of either a fibrotic or a primitive stromal scar. The extent of epithelial injury plays a critical role and determines the stromal response after LASIK or photorefractive keratectomy (PRK) [16]. Studies reported that isolated intrastromal injury involving the anterior cap due to a femtosecond laser prevents epithelial injury and is associated with a favorable wound-healing response preserving corneal transparency [17]. Therefore, predictability after SMILE differs from that of LASIK and PRK.

With the aim of improving the nomograms to increase the predictability of the SMILE procedure, studies focusing on the possible preoperative factors that influence the final outcomes are needed. To our knowledge, few studies have scarcely assessed the effects of preoperative keratometry on visual outcome after SMILE.

The present study investigated the relationship between preoperative keratometry and postoperative refraction and compared the visual outcomes between eyes with preoperative flat and steep keratometric (K) readings for 12 months after SMILE.

## Patients and methods

### Study population

The retrospective review involved patients who underwent SMILE at the Nuri Eye Clinic, Daejeon, South Korea between January 2016 and May 2017. This study was followed the tenets of the Declaration of Helsinki. The Committee of institutional review board was approved all protocols (SCHCA 2020-11-041).

Based on the average keratometry of all patients (814 eyes, 409 patients), two groups were created using a single standard deviation (SD) from the average K, which is 43.21 ± 1.36 D. Of these, 121 eyes of 66 patients with an average K of less than 41.85 D (flat cornea group) were compared with 126 eyes of 73 patients with an average K greater than 44.57 D (steep cornea group).

We also divided the patients into three subgroups according to degrees of spherical equivalent corrected (low myopia: 0.00 to − 2.99 D, moderate myopia: − 3.00 to − 5.99 D, high myopia: − 6.00 to − 9.00 D). All eyes were targeted for emmetropia and follow-up for at least 12 months.

Inclusion criteria included (1) patients who were 18 years of age or older, (2) with no other previous ocular surgery, (3) no current or past ocular pathology, (4) a minimum corneal thickness of 460 µm with minimum estimated residual stromal bed (except cap) of 250 µm after SMILE, (5) no suspicion of keratoconus, (6) stable refraction for 12 months, and (7) Sph ≤ − 9.00 D, Cyl ≤ − 5.00 D.

Exclusion criteria included (1) the presence of residual, recurrent, or active ocular disease such as uveitis, retinal disorder, severe dry eye, or significant cataracts, (2) patients with topographic evidence of forme fruste keratoconus (FFK), and (3) patients with a history of ocular surgery or systemic collagen vascular disease.

### Surgical procedures

All surgical procedures were performed by a single experienced surgeon (S.J.L.). Before the procedure, patients underwent topical anesthesia, standard sterile draping, and speculum insertion.

SMILE was performed using a VisuMax® femtosecond laser (Carl Zeiss Meditec AG, Jena, Germany) with a repetition rate of 500 kHz and set from 110 to 140 nJ pulse energy. The lenticule diameter ranged from5.5 mm to 6.8 mm, and the minimum lenticule thickness from 10 to 15 µm. A 2.0 mm single-sided cut was made in the superior position, followed by dissection of the lenticule and manual removal through a side cut.

To compensate for possible torsional movements with the patient lying supine on the surgical bed, both the eyes were marked preoperatively along the horizontal meridian at the limbus at 3 and 9 o’clock with the patient seated at the slit-lamp.

We used our own nomogram with additional 10% diopter adjustments of the attempted treatment sphere to the attempted sphere: age (years) ≤ 24: *y* = 10% *x* + 0.25, age (years) ≥ 25: *y* = 10% *x*, *x* (D): sphere obtained by manifest refraction, y: attempted sphere.

After SMILE, the postoperative regimen included topical levofloxacin (Levocle®, Hanlim, Seoul, South Korea) administered 4 times daily for 1 week, and a topical loteprednol (Lotemax®, Bausch & Lomb, Tampa, FL, USA) administered 4 times a day for a week. The dosage was gradually reduced over a month.

### Preoperative and postoperative assessments

Before surgery and at 1, 3, 6, and 12 months postoperatively, all patients underwent a detailed ophthalmologic examination that included evaluation of logMAR uncorrected distance visual acuity (UDVA) and corrected distance visual acuity (CDVA), manifest refraction, slit lamp examination (Takagi SM-90 N), measurement of intraocular pressure (noncontact tonometer, NT-530, NCT Nidek Co., Ltd.) and pupil size (Colvard, Oasis Medical), scheimpflug-based corneal topography (Pentacam, Oculus, GmbH), and indirect fundoscopy.

The results were compared between eyes with flat and steep corneas and between eyes with low, moderate, and high myopia.

Keratometry was measured by auto kerato-refractometer (KR-8800, Topcon Corporation). The relationship between mean preoperative keratometry and postoperative spherical equivalent (SE) was analyzed using linear regression.

Refraction stability, predictability and safety were compared between flat and steep corneas at one-year postoperatively. The efficacy index was calculated by dividing postoperative 12 months UDVA by preoperative CDVA. The safety index was calculated by dividing postoperative 12-month CDVA by preoperative CDVA. Predictability represents the proportion of postoperative SE within 0.5 or 1.0 diopters. These indices were calculated using data measured at 12 months postoperatively.

### Statistical analysis

SPSS version 22.0 software (SPSS, Inc., Chicago, IL, USA) was used to perform the statistical analysis. Linear regression analysis and unpaired 2-tailed Student *t* test were performed. A *P* value of 0.05 or less was considered statistically significant. Data are expressed as the mean ± standard deviation.

## Results

The mean age of 409 patients (194 men, 215 women) with 814 eyes was 26.94 ± 7.22 years (range 18–52 years), and the mean preoperative SE refraction was − 5.57 ± 2.03 D (range − 1.00 to − 11.38 D). The mean preoperative keratometry was 43.21 ± 1.36 D (range, 39.87–47.50 D).

In the flat cornea group including 121 eyes, the mean age was 25.30 ± 6.48 years (range 18 to 50 years). Preoperatively, the mean SE refraction was − 5.12 ± 1.96 D (range, − 0.75 to − 9.75 D). The mean keratometry was 41.13 ± 0.52 D (range 39.13–41.75 D), and the mean pachymetry was 541.96 ± 28.29 µm (460–625 µm).

In the steep cornea group including 126 eyes, the mean age was 28.53 ± 7.85 years (range 18–52 years). Preoperatively, the mean SE refraction was − 5.22 ± 2.04 D (range − 1.00 to − 10.13 D), while the mean keratometry was 45.24 ± 0.61 D (range 44.62–47.50 D), and the mean pachymetry was 533.48 ± 29.59 µm (range 480–645 µm).

As shown in Table 1, the refractive results in flat and steep cornea groups for 12 months after SMILE showed no differences. However, the high myopia subgroup revealed an undercorrection of myopia in those with a steep cornea compared with those with low or moderate myopia at 12 months postoperatively (*P* = 0.021).

**Table 1:** Refractive outcomes in flat and steep cornea groups for 12 months after SMILE

	Myopia	Spherical equivalent		*P* value
		Flat K	Steep K	
Preoperation	< − 3	− 2.26 ± 0.71 (*n* = 10)	− 2.39 ± 0.59 (*n* = 18)	0.654
	− 3 ~ − 6	− 4.37 ± 0.81 (*n* = 74)	− 4.39 ± 0.81 (*n* = 62)	0.883
	≥ − 6	− 7.65 ± 1.19 (*n* = 37)	− 7.46 ± 1.13 (*n* = 46)	0.483
1 month	< − 3	0.05 ± 0.23	− 0.18 ± 0.27	0.064
	− 3 ~ − 6	− 0.13 ± 0.36	− 0.03 ± 0.37	0.116
	≥ − 6	− 0.36 ± 0.57	− 0.54 ± 0.46	0.225
	*P* value	0.019	< 0.001	
3 months	< − 3	− 0.07 ± 0.35	− 0.13 ± 0.46	0.689
	− 3 ~ − 6	− 0.17 ± 0.43	− 0.09 ± 0.40	0.312
	≥ − 6	− 0.23 ± 0.52	− 0.47 ± 0.55	0.090
	*P* value	0.559	< 0.001	
6 months	< − 3	− 0.07 ± 0.35	− 0.13 ± 0.23	0.900
	− 3 ~ − 6	− 0.20 ± 0.42	− 0.16 ± 0.37	0.558
	≥ − 6	− 0.35 ± 0.46	− 0.34 ± 0.46	0.617
	*P* value	0.152	0.055	
12 months	< − 3	− 0.23 ± 0.48	− 0.08 ± 0.32	0.869
	− 3 ~ − 6	− 0.19 ± 0.34	− 0.16 ± 0.45	0.611
	≥ − 6	− 0.43 ± 0.58	− 0.44 ± 0.56	0.963
	*P* value	0.102	0.021*	

Correlations between the preoperative keratometry and the pre- and postoperative SE in all patients (*n* = 814 eyes) for the 12 months after SMILE were not significant.

Table 2 shows that the association between preoperative keratometry with postoperative spherical equivalent was only significantly different in flat corneas at 1 month postoperatively. Despite the weak correlation (*r*^2^ = 0.042, *P* = 0.025), this negative regression analysis shows that the flatter the cornea, the higher was the overcorrection in flat corneas 1 month after SMILE.

**Table 2:** Associations of preoperative keratometry with postoperative spherical equivalent in flat and steep corneas

	Flat K			Steep K		
	*ß* ± SD	*R*^2^	*P* value	*ß* ± SD	*R*^2^	*P* value
1 month	− 0.175 ± 0.077	0.042	0.025*	0.008 ± 0.067	0.000	0.908
3 months	− 0.108 ± 0.081	0.015	0.183	− 0.076 ± 0.072	0.009	0.293
6 months	− 0.073 ± 0.086	0.007	0.397	− 0.078 ± 0.058	0.015	0.180
12 months	− 0.065 ± 0.079	0.006	0.411	0.006 ± 0.073	0.000	0.936

*ß*: slope expressed by linear regression analysis, SD: standard deviation

Additionally, the refractive stability in both groups for 12 months was not statistically different and postoperative keratometric changes were not significant between the two groups from 1 to 3 months, 3 to 6 months, and 6 to 12 months.

Table 3 shows that the association of the preoperative keratometry with the postoperative keratometric changes in the flat and steep corneas was significant (*r*^2^ = 0.092, *P* = 0.005 and *r*^2^ = 0.094, *P* = 0.012, respectively) in the 3 to 6 months and 6 to 12 months. The correlations were weakly negative and show that the flatter the cornea, the greater was the postoperative keratometric change in the flat corneas in 3 to 6 months and 6–12 months.

**Table 3:** Association of preoperative keratometry with postoperative keratometric changes in flat and steep corneas

	Flak K			Steep K		
	*ß* ± SD	*R*^2^	*P* value	*ß* ± SD	*R*^2^	*P* value
1—3 months	0.045 ± 0.061	0.006	0.462	− 0.033 ± 0.025	0.004	0.250
3—6 months	− 0.102 ± 0.035	0.092	0.005*	− 0.008 ± 0.30	0.001	0.798
6—12 months	− 0.130 ± 0.05	0.094	0.012*	− 0.050 ± 0.041	0.021	0.217

*ß*: slope expressed by linear regression analysis, SD: standard deviation

Table 4 shows the statistical difference in the relationship between preoperative keratometry and postoperative SE in the low myopia subgroup at one month postoperatively (*r*^2^ = 0.262, *P* = 0.001). The regression analysis shows a negative correlation. The coefficient of determination, r-squared value is below 0.3, suggesting a weak correlation. However, this negative correlation shows the flatter the cornea in the eyes with low myopia, the greater was the postoperative spherical equivalent at one month postoperatively.

**Table 4:** Association of preoperative keratometry with postoperative spherical equivalent according to the degree of myopia

	Low (*n* = 70)			Moderate (n = 438)				High (n = 306)		
Mean preop SE(D)	− 2.20 ± 0.49 (− 1.00 to − 2.88)			− 4.61 ± 0.78 (− 3.00 to − 5.88)				− 7.69 ± 1.31 (− 6.00 to − 11.38)		
	*ß* ± SD	*R*^2^	*P* value	*ß* ± SD	*R*^2^	*P* value		*ß* ± SD	*R*^2^	*P* value
1 month	− 0.132 ± 0.038	0.262	0.001*	0.023 ± 0.020	0.005	0.258		− 0.034 ± 0.030	0.008	0.257
3 months	− 0.050 ± 0.032	0.067	0.123	0.024 ± 0.022	0.005	0.275		− 0.059 ± 0.031	0.021	0.060
6 months	− 0.021 ± 0.038	0.009	0.585	− 0.004 ± 0.022	0.000	0.848		− 0.030 ± 0.026	0.008	0.238
12 months	0.030 ± 0.048	0.010	0.539	0.043 ± 0.022	0.016	0.063		0.014 ± 0.031	0.001	0.646

ß: slope expressed by linear regression analysis, SD: standard deviation

Visual outcomes such as refractive predictability (within ± 0.50 D: 75.2%, 77%, within ± 1.00 D: 95.04%, 90.47%, respectively), efficacy (index: 0.99 ± 0.10, 0.96 ± 0.12, respectively), and safety (index: 1.01 ± 0.06, 1.00 ± 0.05, respectively) between flat and steep corneas at 12 months postoperatively were not significantly different (Table 5).

**Table 5:** Comparative analysis of visual outcomes in flat and steep corneas stratified by the degree of myopia corrected

Degree of myopia (D)	Preoperative Keratometry (D)		*P* Value
	< 41.85 Flat (*n* = 121)	> 44.57 Steep (*n* = 126)	
Low (*n* = 28)			
Number	10	18	−
Mean age (y)	30.14 ± 6.30	28.83 ± 7.28	0.650
Mean SE (D) ± SD			
Preop	− 2.26 ± 0.71	− 2.39 ± 0.59	0.654
Postop	− 0.23 ± 0.48	− 0.08 ± 0.32	0.869
Efficacy	0.98 ± 0.03	0.98 ± 0.03	0.953
Safety	0.91 ± 0.18	0.94 ± 0.13	1.000
Predictability			
% ± 0.5 D (SE)	70	88.9	0.315
% ± 1.0 D (SE)	100	100	
Moderate (*n* = 136)			
Number	74	62	−
Mean age (y)	25.02 ± 5.51	29.97 ± 7.57	0.002*
Mean SE (D) ± SD			
Preop	− 4.37 ± 0.81	− 4.39 ± 0.81	0.883
Postop	− 0.19 ± 0.34	− 0.16 ± 0.45	0.611
Efficacy	0.99 ± 0.06	0.94 ± 0.14	0.035
Safety	1.01 ± 0.04	0.99 ± 0.05	0.117
Predictability			
% ± 0.5 D (SE)	86.5	82.3	0.497
% ± 1.0 D (SE)	98.6	95.2	0.231
High (n = 83)			
Number	37	46	–
Mean age (y)	24.29 ± 7.12	26.96 ± 8.30	0.223
Mean SE (D) ± SD			
Preop	− 7.65 ± 1.19	− 7.46 ± 1.13	0.462
Postop	− 0.43 ± 0.58	− 0.44 ± 0.56	0.938
Efficacy	1.00 ± 0.11	0.98 ± 0.10	0.514
Safety	1.02 ± 0.09	1.02 ± 0.06	0.975
Predictability			
% ± 0.5 D (SE)	54.1	65.2	0.302
% ± 1.0 D (SE)	86.5	80.4	0.464

## Discussion

Previous reports have shown that the SMILE procedure showed predictability, efficiency, stability and safety [1–4]. The predictability and efficiency of excimer laser surgeries, that have been implemented about 30 years ago, is known to be affected by a number of factors, including ablation parallax, corneal hydration, and corneal curvature [18]. However, the SMILE procedure that was first performed 10 years ago induces less corneal epithelial damage than LASIK and PRK, by using a femtosecond laser spherical intrastromal lenticule extraction, which causes less thermal damage to corneal tissues, and varying levels of surgical method and wound recovery compared with LASIK and PRK [16, 17]. Corneal biomechanics and wound healing properties of the cornea undermine the predictability and stability of refractive surgery and contribute to discrepancies between attempted and achieved visual outcomes after LASIK, surface ablation and SMILE [19]. However, the factors affecting visual outcomes after SMILE procedure are still unknown.

In the present study, we demonstrated that visual outcomes such as refractive predictability, efficacy, stability and safety at 12 months postoperatively were not significantly different between preoperative flat and steep corneas. However, eyes with high myopia were less corrected than eyes with moderate and low myopia, especially in steep corneas. These results differ from that of several previous studies [8, 9].

Kim et al.[9] demonstrated the clinical SMILE outcome of high-myopia patients, including efficacy, predictability, and safety, which were comparable to that of patients with mild-to-moderate myopia. In this study, we treated all the eyes with the same nomogram regardless of the degree of keratometry and preoperative myopia, which contributed to an additional 10% diopter adjustment of the attempted treatment spheres. Consequently, there was no difference in the refractive results between flat and steep corneas for 12 months after SMILE, whereas eyes with high myopia were less corrected than eyes with moderate and low myopia, especially in steep corneas. Therefore, when correcting eyes with high myopia, especially in steep corneas, adjustment of the following nomogram is needed. The greater the amount of myopia corrected, the greater is the percentage of myopia that needs to be corrected.

Although the correlations between preoperative keratometry and postoperative spherical equivalent in all patients for the 12 months after SMILE were not significant, the present study shows that the flatter or steeper the cornea in the flat corneas or all the low myopia corneas, the more overcorrected or undercorrected is the postoperative refraction at 1 month postoperatively. Our results are similar to those reported previously suggesting that spherical equivalent refraction undercorrection was predicted by increasing patient age (0.10 D per decade; *P* < 0.01) and steeper corneal curvature (0.04 D per D; *P* < 0.01) [13]. In addition, our study shows that the flatter the cornea, the greater the postoperative keratometric change in flat corneas in 3 to 12 months.

In our study, due to undercorrection of myopia after SMILE, the nomogram was adjusted for the additional myopia requiring correction. A possible reason for undercorrection after SMILE might be associated with that the achieved lenticule diameter is larger than the programmed with the VisuMax femtosecond laser [20]. In addition, as the degree of corrected myopia increased, the degree of undercorrection increased after SMILE due to weaker mechanical and structural properties of the cornea in the higher myopic corneas after surgery, which was linked to more deformable corneal surfaces [21].

Geometrically, in this study, the eyelid pressure in flat corneas exposed to lenticule side cut is less than in steep corneas, so the flatter the cornea, the greater is the postoperative keratometric change in flat corneas between 3 to 12 months. These keratometric changes affect the postoperative refraction for a year after SMILE.

Our study had limitations. Therefore, the findings must be interpreted cautiously. The sample size was small because the patients were divided into three subgroups, limiting confidence in the conclusions.

To summarize, in early phase after SMILE for myopia, the corneas were flatter in the preoperative flat corneas or all the low myopic corneas, and they were more overcorrected. However, most importantly, preoperative corneal curvature does not influence visual outcomes at 1 year after SMILE.

## Data Availability

Data are available on request.

## References

[1] Sekundo W, Kunert KS, Blum M (2011). Small incision corneal refractive surgery using the small incision lenticule extraction (SMILE) procedure for the correction of myopia and myopic astigmatism: results of a 6-month prospective study. Br J Ophthalmol.

[2] Shah R, Shah S, Sengupta S (2011). Results of small incision lenticule extraction: all-in-one femtosecond laser refractive surgery. J Cataract Refract Surg.

[3] Sekundo W, Kunert K, Russmann C (2008). First efficacy and safety study of femtosecond lenticule extraction for the correction of myopia: six-month results. J Cataract Refract Surg.

[4] Blum M, Kunert K, Schröder M (2010). Femtosecond lenticule extraction for the correction of myopia: preliminary 6-month results. Graefes Arch Clin Exp Ophthalmol.

[5] Vestergaard A, Ivarsen A, Asp S (2013). Femtosecond (FS) laser vision correction procedure for moderate to high myopia: a prospective study of ReLEx® flex, and comparison with a retrospective study of FS-laser in situ keratomileusis. Acta Ophthalmol.

[6] Vestergaard A, Ivarsen A, Asp S (2012). Small-incision lenticule extraction for moderate to high myopia: predictability, safety, and patient satisfaction. J Cataract Refract Surg.

[7] Stodulka P (2019) Correction of hyperopia with hyperopic astigmatism by ReLEx SMILE: a case report. In: Proceedings of the ESCRS winter meeting. Athens, Greece

[8] Chansue E, Tanehsakdi M, Swasdibutra S (2015). Efficacy, predictability and safety of small incision lenticule extraction (SMILE). Eye Vis (Lond).

[9] Kim JR, Kim BK, Mun SJ (2015). One-year outcomes of small-incision lenticule extraction (SMILE): mild to moderate myopia vs. high myopia. BMC Ophthalmol.

[10] Rao SK, Cheng AC, Fan DS (2001). Effect of preoperative keratometry on refractive outcomes after laser in situ keratomileusis. J Cataract Refract Surg.

[11] Cobo-Soriano R, Llovet F, González-López F (2002). Factors that influence outcomes of hyperopic laser in situ keratomileusis. J Cataract Refract Surg.

[12] de Benito-Llopis L, Teus MA, Sánchez-Pina JM (2008). Influence of preoperative keratometry on refractive results after laser-assisted subepithelial keratectomy to correct myopia. J Cataract Refract Surg.

[13] Hjortdal JØ, Vestergaard AH, Ivarsen A (2012). Predictors for the outcomes of small-incision lenticule extraction for myopia. J Refract Surg.

[14] Blaker JW, Hersh PS (1994). Theoretical and clinical effect of preoperative corneal curvature on excimer laser photorefractive keratectomy for myopia. J Refract Corneal Surg.

[15] Christiansen SM, Neuffer MC, Sikder S (2012). The effects of preoperative keratometry on visual outcomes after moderate myopic LASIK. Clin Ophthalmol.

[16] Netto MV, Mohan RR, Ambrosio R (2005). Wound healing in the cornea: a review of refractive surgery complications and new prospects for therapy. Cornea.

[17] Meltendorf C, Burbach GJ, Bühren J (2007). Corneal femtosecond laser keratotomy results in isolated stromal injury and favorable wound-healing response. Invest Ophthalomol Vis Sci.

[18] Kim WS, Jo JM (2001). Corneal hydration affects ablation during laserin situ keratomileusis surgery. Cornea.

[19] Dupps WJ, Wilson SE (2006). Biomechanics and wound healing in the cornea. Exp Eye Res.

[20] Reinstein DZ, Pradhan KR, Carp GI (2017). Small incision lenticule extraction (SMILE) for hyperopia: optical zone diameter and spherical aberration induction. J Refract Surg.

[21] Wu W, Wang Y (2015). The correlation analysis between corneal biomechanical properties and the surgically induced corneal high-order aberrations after small incision Lenticule extraction and femtosecond laser in situ Keratomileusis. J Ophthalmol.

